# Identification and Characterization of Epstein-Barr Virus Genomes in Lung Carcinoma Biopsy Samples by Next-Generation Sequencing Technology

**DOI:** 10.1038/srep26156

**Published:** 2016-05-18

**Authors:** Shanshan Wang, Hongchao Xiong, Shi Yan, Nan Wu, Zheming Lu

**Affiliations:** 1 Key Laboratory of Carcinogenesis and Translational Research, Ministry of Education, Laboratory of clinical laboratory, Peking University Cancer Hospital and Institute, Beijing, China; 2 Department of Thoracic Surgery, Peking University Cancer Hospital and Institute, Beijing, China; 3 Laboratory of Genetics, Peking University Cancer Hospital and Institute, Beijing, China

## Abstract

Epstein-Barr virus (EBV) has been detected in the tumor cells of several cancers, including some cases of lung carcinoma (LC). However, the genomic characteristics and diversity of EBV strains associated with LC are poorly understood. In this study, we sequenced the EBV genomes isolated from four primary LC tumor biopsy samples, designated LC1 to LC4. Comparative analysis demonstrated that LC strains were more closely related to GD1 strain. Compared to GD1 reference genome, a total of 520 variations in all, including 498 substitutions, 12 insertions, and 10 deletions were found. Latent genes were found to harbor the most numbers of nonsynonymous mutations. Phylogenetic analysis showed that all LC strains were closely related to Asian EBV strains, whereas different from African/American strains. LC2 genome was distinct from the other three LC genomes, suggesting at least two parental lineages of EBV among the LC genomes may exist. All LC strains could be classified as China 1 and V-val subtype according to the amino acid sequence of LMP1 and EBNA1, respectively. In conclusion, our results showed the genomic diversity among EBV genomes isolated from LC, which might facilitate to uncover the previously unknown variations of pathogenic significance.

Epstein-Barr virus (EBV) is a lymphotrophic herpesvirus infecting more than 90% of the adults worldwide^1^. It has been implicated in the pathogenesis of a variety of human lymphoid and epithelial malignancies, including Burkitt’s lymphoma (BL), Hodgkin lymphoma (HL), nasopharyngeal carcinoma (NPC), and EBV-associated gastric carcinoma (EBVaGC)^2^,^3^. The association of EBV and lung carcinoma (LC) presents significant differences according to tumor histotype and geographical site^4^. EBV is often detected in Lymphoepithelioma-like carcinoma (LELC) of the lung in Asian patients^5^,^6^,^7^, while is sporadic detected in other types of lung carcinoma according to previously published small series and case reports^7^,^8^,^9^,^10^. Geographic location and different tumor histotype may associate with the presence of certain EBV strains. Characterizing the sequences and variations of infecting EBV would facilitate to understand their potential roles in LC pathogenesis, and would contribute to the development of therapeutic approaches in the future^11^. Genetic variations of EBV have been invested by genotyping polymorphic markers in small subsets of genes or by whole genome sequencing and comparison analysis in EBVaGC and NPC^12^,^13^,^14^,^15^,^16^,^17^,^18^,^19^. However, the EBV genomic variations in lung carcinoma have not been systematically explored.

More than 100 EBV strains have been completely or partially sequenced to date: B95-8, AG876, Akata, Mutu, GD1, GD2, C666-1, K4413-Mi, K4123-Mi, nine NPC EBV sequences HKNPC1 to -9, nine EBVaGC sequences EBVaGC1 to -9, and 71 EBV genomes from different sample types and locations worldwide, including spontaneous lymphoblastoid cell lines (LCLs) from Australia and Kenya, Burkitt lymphoma cell lines, Hodgkin lymphoma primary biopsies and cell lines, NPC cell lines and biopsy, one gastric cancer cell line and one EBV strain from the saliva of a healthy individual^16^,^17^,^18^,^19^,^20^,^21^,^22^,^23^,^24^. B95-8 was the first completely sequenced EBV genome derived from a North American case of infectious mononucleosis^25^. A more complete 171 kb wild-type EBV (EBV-WT) reference genome was constructed using B95-8 as a backbone with an 11 kb missing fragment provided by Raji sequence^26^. Akata and Mutu were derived from Burkitt’s lymphomas and sequenced by next-generation sequencing (NGS)^20^. K4413-Mi and K4123-Mi were sequenced from immortalized human B lymphocyte cell lines^23^. C666-1 was derived from a native EBV-infected NPC cell line of southern Chinese origin^24^. GD1, GD2, and HKNPC1 to -9 were all EBV genomes derived from NPC patients. Specifically, GD1 was isolated from the saliva of a NPC patient and sequenced using conventional shotgun sequencing^19^, while GD2 and HKNPC1 to -9 were isolated from primary NPC biopsy specimens and sequenced using NGS^16^,^17^,^18^. EBVaGC1 to -9 were isolated from EBVaGC biopsy specimens^21^. AG876 originated from a Ghanaian case of Burkitt’s lymphoma and was the first complete type 2 EBV sequence^27^. Most recently, 11 more type 2 EBV sequence were sequenced by NGS^22^. However, there was no complete EBV genome sequence derived from lung carcinoma reported yet.

In this study, we detected the presence of EBV in primary lung carcinomas by EBER *in Situ* hybridization (ISH). Subsequently, we performed EBV genomes capture, next-generation sequencing, *de novo* assembly, and joining of contigs by Sanger sequencing. The sequences of four LC biopsy specimen-derived EBV (LC-EBV) genomes were then determined. Furthermore, comparative and phylogenetic analyses were performed to assess the genomic diversity among the LC-EBV genomes.

## Results

### EBER expressed in lung carcinoma

**Figure 1:**
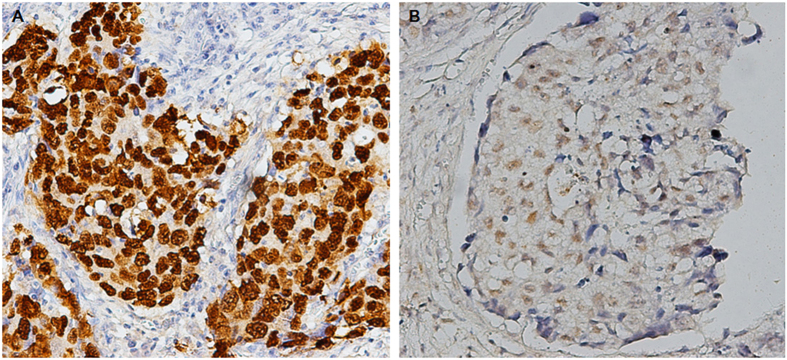
*In situ* hybridization for EBER. (**A**) Squamous carcinoma associated with EBV. *In situ* hybridization for EBER shows the brown-stained nuclei in squamous-cell nests, ×200. (**B**) Adenocarcinoma associated with EBV. Note the brown-stained nuclei in most of tumor cells, ×200.

EBV was detected in four out of 66 lung carcinoma cases by ISH staining for EBER. EBER staining was confined to the nuclei of carcinoma cells, but not in surrounding non-neoplastic cells (Fig. 1). The clinicopathological features of four cases are summarized in supplementary Table S1. The positive cases included three men and one woman, whose ages ranged from 57 years to 77 years. Histologically, one case was adenocarcinoma and three cases were squamous carcinomas.

### Sequencing and assembly of LC-EBV genomes

All of four LC-EBV genomes were successfully captured and sequenced. Sequence reads that passed default quality control filters on the Illumina platform were aligned to the six reported EBV genomes (EBV-WT, AG876, B95-8, GD1, GD2, and HKNPC1). The coverage of aligned reads on GD1 genome was 96.1%, 98.2%, 97.7%, and 91.5%, respectively, which was highest among the six EBV genomes. Therefore, GD1 was served as the reference genome for the subsequent analyses. The percentage of mapped reads for LC1 to -4 was 27.9%, 29.8%, 24.9%, and 27.5%, respectively. The mean coverage for the LC1 to -4 genomes was 423-fold (supplementary Table S2).

The human sequences were first removed, and the remaining reads were *de novo* assembled into scaffolds using Velvet. The number of contigs for LC1 to -4 was 20, 20, 25, and 20, respectively. N50 sizes of contigs ranged from 16,795 bp (LC1) to 19,803 (LC2). The longest contigs were ~44 kb in length for all of the samples. A summary of the assembled sequences and the contig sizes is given in supplementary Table S3. The gaps between the contigs were filled up by the sequence derived from PCR and Sanger sequencing or by tracts of “N” with length estimated based on the EBV reference GD1. Finally, four EBV genomes were determined, designated LC1 to LC4. The genome sizes estimated based on the reference EBV sequence were as follows: LC1 (171,563 bp), LC2 (171,649 bp), LC3 (171,742 bp) and LC4 (171,605 bp), with GC-contents of ~56%. The percentage of the genome that is represented by tracts of “N” for each strain was 4.26% (LC1), 4.49% (LC2), 4.42% (LC3) and 4.38% (LC4), respectively. For validation and gap filling, the EBNA1 and LMP1 genes were amplified and sequenced by conventional Sanger sequencing. The sequences determined by Sanger sequencing were identical to those of the assembly, suggesting the high-confidence level of the assembled sequences.

### Mutation analysis of the LC genomes

**Figure 2:**
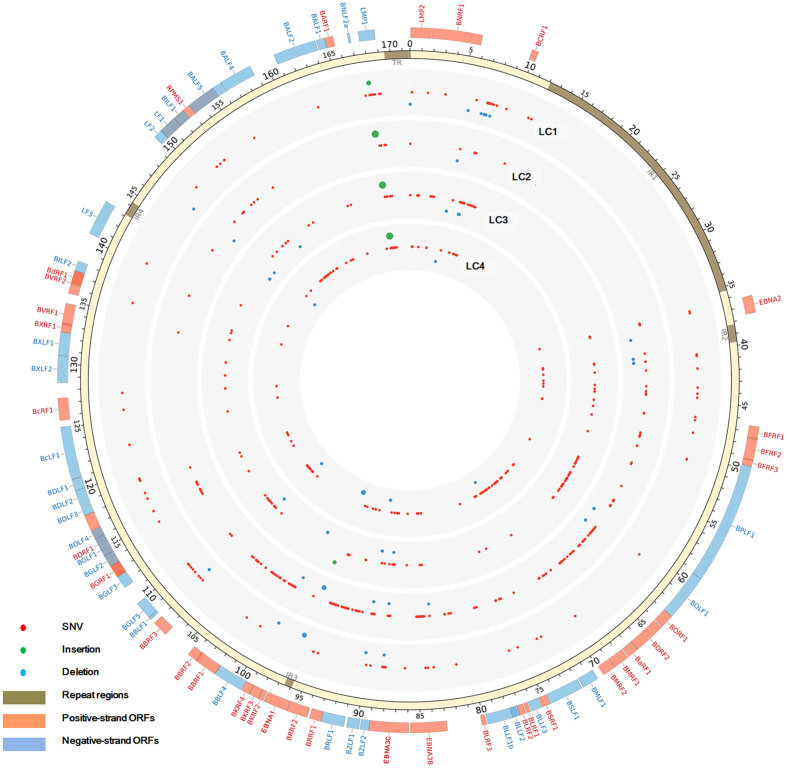
Genetic variations among LC strains. Circos plot demonstrates genetic variations of LC strains relative to the reference GD1 strain (AY961628). Mutations in internal repeats and terminal repeats are disregarded. The outer circle shows the positive-strand open reading frames (ORFs) (orange), repeat regions (brown), and negative-strand ORFs (blue) in the reference GD1 genome. The red, blue, and green points in the inner circles show the distributions of single nucleotide variations (SNVs), deletions, and insertions, respectively. The size of the point represents the length of the deletion or insertion.

Compared to the reference genome GD1, a total of 520 variations in all, including 498 substitutions, 12 insertions, and 10 deletions were found in the LC1 to -4 genomes. Among them, 363 substitutions, 7 insertions, and 5 deletions were located in the coding regions of the genomes, while 135 substitutions, 5 insertions, and 5 deletions were found in the noncoding regions. A summary of the variations in LC-EBV genomes is given in supplementary Table S4. The variability of the LC-EBV genomes, which was calculated by dividing the number of variations by the total number of bases of genomes, ranged from 0.059% (LC1) to 0.150% (LC2). Figure 2 illustrates the variations of all LC-EBV genomes relative to the reference EBV strain GD1.

**Figure 3:**
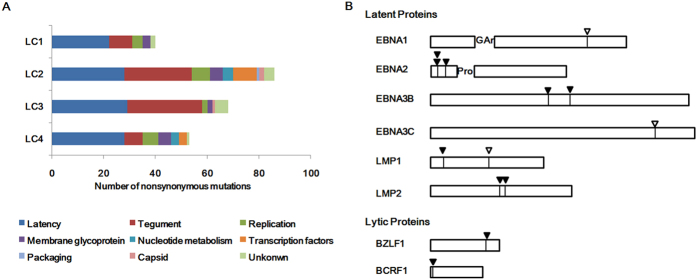
Nonsynonymous mutations of LC strains. (**A**) Number of nonsynonymous mutations contained in the nine categories of EBV proteins. The majority of the amino acid changes are located in latent proteins (blue) in all of the LC strains, followed by tegument proteins (red). The Latency category refers to all latent proteins expressed in EBV virus latent phase, whereas all other categories refer only to lytic proteins. (**B**) Amino acid changes in CD8^+^ and CD4^+^ T cell epitopes. Amino acid changes in at least one of the LC strains at known CD8^+^ and CD4^+^ T cell epitopes are marked with solid and hollow arrows, respectively.

EBV proteins can be classified to nine categories according to function^28^. Latent genes in all of the LC-EBV genomes were found to harbor the highest numbers of nonsynonymous mutations, followed by tegument genes (Fig. 3A). Latent genes contained 22 (55%), 28 (32.6%), 29 (42.6%), and 28 (52.8%) nonsynonymous mutations in LC1 to -4 genomes. Genes encoding tegument proteins contained 9 (22.5%), 26 (30.2%), 29 (42.6%), and 7 (13.2%) nonsynonymous mutations in LC1 to -4 genomes. LC2 had the highest number of 7 (8.1%) nonsynonymous mutations in genes encoding proteins for replication. The remaining nonsynonymous mutations were located in genes encoding proteins for replication, membrane glycoproteins, transcription, capsid, packaging, nucleotide metabolism or in proteins of unknown function.

### Amino acid changes in CD4^+^ and CD8^+^ T-cell epitopes identified in EBV lytic and latent proteins

According to the CD4^+^ and CD8^+^ T-cell epitopes defined and reviewed in previous publications, amino acid changes in latent and lytic proteins derived epitopes were examined^29^,^30^. Compared to GD1, amino acid changes were found in two CD8^+^ epitopes of EBNA2, two epitopes of EBNA3B, two epitopes of LMP2, and one epitope of LMP1, BZLF1, and BCRF1, respectively. EBNA1, -3C, and LMP1 proteins harbor one amino acid change in the CD4^+^ epitope respectively. A T-to-C substitution at 1134 resulted in the change of residue 251 (I-to-T) in LMP2, where CD8^+^ epitopes TVC and MFI were located. A T-to-C substitution at 84175 resulted in the change of residue 399 (A-to-P) in EBNA3B protein, where HLA A11-restricted immunodominant epitope AVF was locate^31^. The positions of the amino acid changes located in the epitopes are illustrated in Fig. 3B and tabulated in supplementary Table S5.

### EBNA1 and LMP1 variations

Based on the signature changes at amino acid (AA) residue 487 in the carboxyl-terminal of EBNA1, EBV has been classified into five subtypes, including P-ala, P-thr, V-leu, V-val, and V-pro^13^,^32^. All of the four LC strains were identified as V-val subtype, which was detected in both cases and controls almost exclusively in China^1^. Compared to the EBNA1 sequence of EBV-WT, 15 nonsynonymous mutations were shared amongst LC1 to -4 and GD1. Only one amino acid substitution was found (Ile at Thr585) in LC2 compared to GD1.

**Figure 4:**
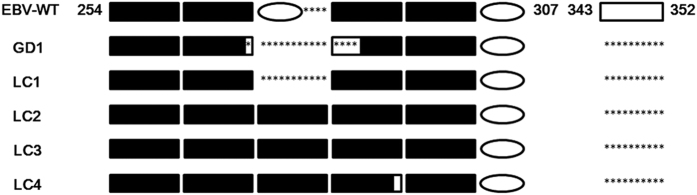
Schematic diagram of variations in the 11-AA repeat and 10-AA deletion in the C-terminus of LMP1. The pattern of the EBV-WT is shown across the top with the number indicating the amino acid (AA) positions of both sides. A black rectangle represents 11-AA (QDPDNTDDNGP) repeat element. A black rectangle with a blank window or asterisks represents 11-AA repeat element with single nucleotide variation or deletion. A blank oval represents 5-AA (HDPLP) insertion. A blank rectangle represents 10-AA (GGGHSHDSGH). Asterisks denote deletion.

According to the LMP1 sequence, LC strains were differentiated as China 1 type, which is the most prevalent type in Asia^1^,^33^. The N-terminal cytoplasmic tail and transmembrane domain displayed complete conservation among LC strains, while the CTARs demonstrated more diversity with two amino acids that were not present in previously published EBV sequences, including the substitution Tyr at Asn251 observed in LC4 and the substitution Gln at His308 observed in LC1. Compared to EBV-WT, a 30-bp deletion causing a loss of 10-amino acid (AA 343 to 352) in the C-terminus of LMP1 was shared among all LC strains, HKNPCs and EBVaGCs except EBVaGC6 (Fig. 4). Another sequence length variant, resulting from an 11-AA (QDPDNTDDNGP) repeat element with varying copy numbers existed between AA254 and AA307 in the C-terminal domain of LMP1 in LC genomes. The number of repeated sequences varied from four to five repeats, and the five-AA insertion (HDPLP: 276–280) existing between the second and third repeat in EBV-WT was not detected in the middle of the repeats in LC isolates. LC4 had a substitution of Ser at Gly in the third repeat which was unique among the LC isolates.

### Phylogenetic analysis of the LC-EBV genomes

**Figure 5:**
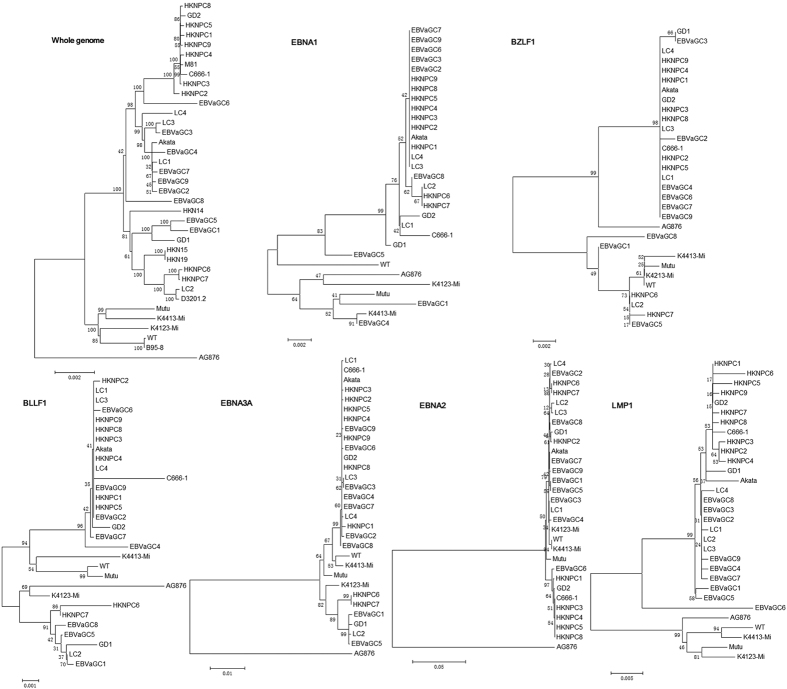
Phylogenetic analysis of the whole EBV genomes and protein-encoding nucleotide sequences of LMP1, EBNA1, -2, -3A, BZLF1, and BLLF1 genes. Phylogenetic analysis was performed using MEGA software (version 6.0) by Neighbour-joining (NJ) algorithm on the basis of multiple alignment of EBV strains. Bootstrap values are shown at the internal nodes.

The phylogenetic tree was constructed based on whole-genome alignment of four LC-EBV genomes and previously published EBV genomes. The result showed that all LC-EBV were clustered with the Asian EBV strains, including HKNPC1 to -9, EBVaGC1 to -9, HKN14, HKN15, HKN19, D3201.2, GD1, GD2, C666-1, and Akata (Japanese strain), while non-Asian strains AG876, B95-8, Mutu, K4413-Mi, and K4123-Mi were clustered to another branch. Similar results were observed when the sequences of LMP1 gene were compared. In addition, LC1, LC3, and LC4 showed a closer distance, while LC2 was clustered to a different branch with HKNPC6, -7 and EBVaGC1, -5 when the nucleotide sequences of EBNA3A and EBV lytic genes BZLF1 and BLLF1 were analyzed (Fig. 5). Analyses on the sequences of EBNA2 and EBNA3A genes showed that all LC genomes are type 1 viruses. Phylogenetic analysis of nucleotide sequences of the EBNA1 gene showed that LC1 was located in a branch distinct from that of the LC2 to -4 strains.

## Discussion

The association of lung carcinoma and EBV showed significant differences according to tumor histotype and geographical site^4^. In the current study, EBV was detected by EBER ISH in one adenocarcinoma and three squamous carcinomas of lung in northern China (Fig. 1). Combined with the reported cases of EBV-associated non-LELC of the lung^7^,^8^,^9^,^10^, these findings support the idea that the EBV-associated lung carcinomas are not restricted to the typical LELC.

The large EBV genome combined with the relative small quantity of viral DNA in the tumor sample presents a sequencing challenge^22^. Therefore we performed selective capture EBV DNA using probes covering genomes of six strains including EBV-WT, AG876, B95-8, GD1, GD2, and HKNPC1. The average percentage of reads for LC1 to -4 that mapped to GD1 genome sequence was 27.5%, suggesting the effective enrichment of EBV DNA by the capture procedure. By *de novo* assembly and contig joining using Sanger sequencing, four EBV genomes were constructed (Fig. 2).

Whole-genome sequencing of EBV in the lung carcinoma enabled the comparison and determination of EBV variations at the genome level. Consistent with previous reports^17^,^22^, we observed the highest number of nonsynonymous mutations in latent genes among LC genomes, followed by genes encoding the tegument proteins and membrane glycoproteins (Fig. 3). Some of these mutations found in lytic and latent genes resulted in amino acid changes in the immune epitopes. For example, a A-to-P amino acid changes of immunodominant epitope AVF in EBNA3B protein were found in LC2 genomes, which was reported to be poor recognized by AVF-specific cytotoxic T cells^31^ and thus might contribute to the evasion of the EBV-infected cells from T cell surveillance.

All of four LC strains could be classified as China 1 according to the amino acid sequence of LMP1. The 30-bp deletion resulting in 10-AA loss in LMP1 was detected in all LC strains (Fig. 4), HKNPCs and EBVaGCs except EBVaGC6. Previous studies have reported different frequencies of the 30-bp deletion in several malignancies in Asia. Tan *et al.* showed 84% of NPC biopsy tissues has the 30-bp deletion in Malaysia^34^. The prevalence of this deletion in NPC was 51.6% in Vietnam^35^. A high prevalence of 30-bp deleted LMP1 was reported in nasal NK/T-cell lymphoma from Malaysia (100%)^36^, 91% in Hong Kong^37^, and 86% in Taiwan, while 81.5% of the EBV-positive control reactive lymphoid tissues also had the 30-bp deleted LMP1 in Taiwan. The prevalence of 30-bp deletion in the LMP1 was lower in samples from Africa, North America and Europe^1^. These results suggested that 30-bp deletion of LMP1 represents a geographic or race associated polymorphism rather than a disease phenotype-associated polymorphism^38^.

Based on the signature codon 487 as well as particular amino acid alterations in other sites of EBNA1, previous studies identified V-val subtype was the dominant subtype in various EBV-positive samples (lymphoma, NPC, EBVaGC, and healthy donors) in Asian regions irrespective of NPC-epidemicity, whereas was rarely found in non-Asian regions^1^,^13^,^39^,^40^,^41^. Chen *et al.* reported V-val EBNA1 subtypes in 25/25 cases of EBV-positive gastric carcinoma and in 8/8 cases of EBV-positive reactive lymphoid follicular hyperplasia in Japanese patients^41^. Wang *et al.*^13^ also found that V-val subtype was prevalent in EBVaGC and throat washing samples of healthy donors in Shandong Province, northern China. It is not surprising that all of the LC strains from Beijing in Northern China were V-val variants, consistent to the findings in different EBV-positive samples in the same area^42^. This result provided another piece of evidence that V-val represents a dominant EBNA1 subtype in Asian regions.

The phylogenetic analysis based on whole-genome alignment of four LC genomes and published EBV genomes showed that the Asian isolates, LC1 to -4, HKNPC1 to -9, EBVaGC1 to -9, HKN14, HKN15, HKN19, D3201.2, GD1, GD2, C666-1, and Akata, formed one relatively compact cluster, while the African/American isolates, AG876, B95-8, and Mutu, clustered to another branch (Fig. 5). This result suggested that geographical distribution factor may be a dominant driver of sequence variations. Similar results were observed from the alignments of nucleotide sequence of LMP1, consistent with the previous report^22^, suggesting that LMP1 gene can serve as a geographical marker. Phylogenetic trees for BZLF1, BLLF1, EBNA3A nucleotide sequences, and the whole-genome sequences showed a closer distance among LC1, LC3, and LC4, while LC2 was clustered into a different branch with HKNPC6 and -7, which were both isolated from advanced metastatic NPC cases, suggesting at least two parental lineages of EBV among the LC genomes may exist.

In summary, we reported four newly sequenced EBV genomes isolated from primary lung carcinomas and demonstrated the genomic diversity among these EBV genomes. Further studies should be performed to assess whether EBV genomic variations contribute to LC pathogenesis.

## Materials and Methods

### LC patients

Lung carcinoma cases were collected from Beijing Cancer Hospital, Beijing, China. All experiments were performed in accordance with relevant guidelines and were approved by the medical ethics committee of the Beijing Cancer Hospital & Institute for Medical Research Ethics. All patients have given informed consent for the use of material for research purposes.

Among the 66 cases, 32 (48.5%) cases were male and 34 cases (51.5%) were female. The mean age was 59.0 ± 11.6 years (range: 31–81 years) for all patients, 61.3 ± 10.1 years (range: 36–81 years) for the male patients, and 56.8 ± 12.7 years (range: 31–79 years) for the female patients.

### *In Situ* hybridization for EBER

The presence of EBV was examined by ISH using EBV oligonucleotide probes complementary to the EBER (Leica Biosystems Newcastle Ltd, Newcastle Upon Tyne, United Kingdom) according to the manufacturer’s instructions. A positive reaction was characterized by intense brown nuclear staining under a light microscopy.

### Sample DNA preparation

Fresh LC tumor biopsy specimen was temporarily stored in phosphate-buffered saline with 1% fetal bovine serum, and DNA was isolated using a Qiagen blood and tissue kit according to the manufacturer’s protocol (Qiagen, Hilden, Germany) within one hour after incision. A NanoDrop spectrophotometer (Thermo Scientific, DE, USA) was used to determine the concentration of the DNA samples. Nondegraded DNA with an A260/A280 ratio between 1.8 and 2.0 was used for the subsequent experiments.

### EBV probes design and EBV genome enrichment and sequencing

Each sequenced sample was prepared according to the instruction of Illumina protocols. Briefly, 3 μg of genomic DNA was sheared to around 150 bp DNA fragments by Covaris S2 (Covaris, Inc., Woburn, MA). DNA fragments were purified, end blunted, “A” tailed, adaptor ligated, size selected, and amplified by 7 cycles of PCR. The concentration of libraries was quantified by NanoDrop spectrophotometer (Thermo Scientific, DE, USA). Full-length EBV genomes of 6 strains, including EBV-WT (NC_007605), AG876 (DQ279927), B95-8 (V01555), GD1 (AY961628), GD2 (HQ020558), and HKNPC1 (JQ009376) were used to design the EBV probes by MyGenostics (MyGenostics, Beijing, China). The capture experiment was conducted according to manufacturer’s protocol. In brief, libraries were hybridized with EBV probes at 65 °C for 24 hours and then washed to remove uncaptured fragments. The eluted fragments were amplified by 14 cycles of PCR to generate libraries for sequencing. Libraries were quantified and preceded to sequencing for paired-end 125 bp using the Illumina Hiseq2500 sequencer according to manufacturer’s instructions (Illumina Inc., San Diego, CA, USA).

### *De novo* assembly of EBV Genomes

For the quality control, the low quality reads were filtered out using the Trim Galore program, and then 3′/5′ adapters were trimmed using the Cutadapt program implemented in Trim Galore. Only reads which sequencing quality is greater than 20 and read length is greater than 80 bp were retained. The high quality reads were aligned to human genome (NCBI build 37, hg19) and each reference EBV genomes (EBV-WT, AG876, B95-8, GD1, GD2, and HKNPC1) using Burrows-Wheeler Aligner (BWA) software (version 0.5.8c, default settings). After human sequences were removed, the remaining reads were assembled using Velvet software (Version 1.2.10). The settings were optimized for each sample using the k-mer lengths of 59 to 73. Subsequently, the contigs were analyzed by BLAST using the NCBI nonredundant nucleotide (NT) database to identify the location and orientation. GD1 was served as the reference genome because of the highest coverage of GD1 genome among all EBV strains. Finally, the gaps were filled up by PCR amplification and conventional Sanger sequencing using the primer sets listed in supplementary Table S6. The regions failed to be amplified were filled by tracts of “N” with length estimated based on reference EBV genome GD1. The same copy number of internal repeat 1 to that of the reference EBV genome was adopted for all of the sequenced LC genomes.

### Identification of variations in the LC genome sequences

Single nucleotide variations (SNVs) and insertions and deletions (indels) were called using the Genome Analysis Toolkit (GATK v2.8). Briefly, duplicated reads were removed using Sequence Alignment/Map tools (SAMtools) 3 and only uniquely mapping reads were used for variation detection. SNVs were detected and genotyped with the GATK UnifiedGenotyper in single-sample mode (with parameters -im ALL -mbq 20 -mmq 20 -mm42 3 -deletions 0.05). Variants were filtered with GATK VariantFiltration module (with filters “QUAL < 50.0 & QD < 5.0 &HRun > 10 & DP < 4” and parameters -cluster 3 -window 10). Indels were detected with GATK IndelGenotyperV2 (with parameters -im ALL) and filtered with a custom python module that removed sites with amax_cons_av ≥ 1.9 (maximum average number of mismatches across reads supporting the indel) or max_cons_nqs_av_mm ≥ 0.2 (maximum average mismatch rate in the 5-bp NQS window around the indel, across indel-supporting reads).

### Phylogenetic analysis

The MUSCLE (Multiple Sequence Comparison by Log-Expectation) program (version 3.52) was applied to perform multiple sequence alignments with default parameters. Phylogenetic analysis of whole genomes of EBV strains was performed using the neighbor-joining (NJ) algorithm implemented in Molecular Evolutionary Genetics Analysis (MEGA) software (version 6.0). Phylogenetic analyses on LMP1, EBNA1, and BZLF1 were also conducted. The reliability of the tree was tested using a bootstrapping method with 1000 replicates.

### Accession numbers

Sequence data for the four LC-EBV genomes were submitted to the GenBank database under accession numbers KT823506 (LC1), KT823507 (LC2), KT823508 (LC3), and KT823509 (LC4). Raw sequencing data were submitted to the Sequence Read Archive (study accession number PRJNA297136).

## Additional Information

**How to cite this article**: Wang, S. *et al.* Identification and Characterization of Epstein-Barr Virus Genomes in Lung Carcinoma Biopsy Samples by Next-Generation Sequencing Technology. *Sci. Rep.* **6**, 26156; doi: 10.1038/srep26156 (2016).

## Supplementary Material

Supplementary Information
