# Supplementary material for: Identification and Characterization of Epstein-Barr Virus Genomes in Lung Carcinoma Biopsy Samples by Next-Generation Sequencing Technology
Source: Sci Rep. 2016 May 18;6:26156. doi: 10.1038/srep26156 (PMC4870493; doi:10.1038/srep26156)
Supplement: Supplementary Information [file srep26156-s1.pdf]

**Identification and Characterization of Epstein-Barr Virus Genomes in Lung Carcinoma Biopsy Samples by Next-Generation Sequencing Technology**

Shanshan Wang, Hongchao Xiong, Shi Yan, Nan Wu, Zheming Lu

**Supplementary Information**

**Table S1. Clinicopathological characteristics of lung carcinomas**

| Case | Age(years) | Gender | Histologic type | Stage | EBV status EBER-1 |
|---|---|---|---|---|---|
| LC1 | 76 | male | squamous carcinoma | T2aN0M0 | + |
| LC2 | 77 | male | squamous carcinoma | T1bN0M0 | + |
| LC3 | 60 | male | squamous carcinoma | T2aN0M0 | + |
| LC4 | 57 | female | adenocarcinoma | T2aNxM0 | + |

**Table S2. Summary of the sequencing data**

| Sample | Total effective yield (Mb) | Mappable reads (% ) | Coverage of GD1 genome (%) | Avg coverage (fold) |
|---|---|---|---|---|
| LC1 | 237.97 | 27.9 | 96.1 | 386.79 |
| LC2 | 283.88 | 29.8 | 98.2 | 492.93 |
| LC3 | 445.7 | 24.9 | 97.7 | 647.8 |
| LC4 | 102.66 | 27.5 | 91.5 | 164.34 |

**Table S3 Summary of contigs assembled from HiSeq**

| Sample | Contig Number | Total Length （bp） | N50 | Shortest （bp） | Longest （bp） |
|---|---|---|---|---|---|
| LC1 | 20 | 143146 | 16795 | 241 | 43711 |
| LC2 | 20 | 142352 | 19803 | 227 | 44055 |
| LC3 | 25 | 144075 | 19773 | 205 | 42564 |
| LC4 | 20 | 141848 | 16899 | 241 | 44219 |

**Table S4 Summary of variations in LC-EBV compared to GD1**

| | | Total | Coding region | Non-coding region | Non-synouymous |
|---|---|---|---|---|---|
| **LC1** | Substitution | 93 | 65 | 28 | 37 |
| | Insertion | 6 | 2 | 4 | / |
| | Deletion | 3 | 1 | 2 | / |
| **LC2** | Substitution | 244 | 189 | 55 | 79 |
| | Insertion | 9 | 5 | 4 | / |
| | Deletion | 4 | 2 | 2 | / |
| **LC3** | Substitution | 159 | 127 | 32 | 64 |
| | Insertion | 6 | 4 | 2 | / |
| | Deletion | 4 | 1 | 3 | / |
| **LC4** | Substitution | 172 | 107 | 65 | 51 |
| | Insertion | 5 | 2 | 3 | / |
| | Deletion | 1 | 0 | 1 | / |

**Table S5 Amino acid changes in $CD8^+$ and $CD4^+$ T-cell epitopes**

| Amino acid changes in EBV-encoded CD8+ T cell-specific epitopes | | | | | | |
|---|---|---|---|---|---|---|
| **Gene** | **Amino acid sequence** | **HLA** | **LC1** | **LC2** | **LC3** | **LC4** |
| **EBNA2 (487aa)** | | | | | | |
| 14-23 | YHLIVDTDSL | B39 | | 4th I>L | 4th I>L | |
| | YHLIVDTDSV | B38 | 10th V>L | 10th V>L | 10th V>L | 10th V>L |
| 42-51 | DTPLIPLTIF | A2/B51 | | 10th F>I | 10th F>I | |
| **EBNA3B (938aa)** | | | | | | |
| 399-408 | AVFDRKSDAK | A11 | | 1st A>P | | |
| 488-496 | TVLLHEESM | B35.01 | | 1st T>A | | |
| **LMP1 (386aa)** | | | | | | |
| 38-46 | FWLYIVLSD | | 7th L>M | 7th L>M | 7th L>M | 7th L>M |
| **LMP2 (497 aa)** | | | | | | |
| 243-251 | TVCGGIMFI | A1 | | | 9th I>T | |
| 249-262 | MFIACVLVLIVDAV | | | | 3rd I>T | |
| **BZLF1 (245 aa)** | | | | | | |
| 190-197 | RAKFKHLL | B8 | | 6th H>Q | | |
| **BCRF1 (170 aa)** | | | | | | |
| 3-11 | RRLVVVLQC | B27 | | | | 6th V>M |

| Amino acid changes in EBV-encoded CD4+ T cell-specific epitopes | | | | | | |
|---|---|---|---|---|---|---|
| **Gene** | **Amino acid sequence** | **HLA** | **LC1** | **LC2** | **LC3** | **LC4** |
| **EBNA1 (641aa)** | | | | | | |
| 574-593 | VLKDAIKDLVMTKPAPTCNI | | | 12th T>I | | |
| **EBNA3C (992aa)** | | | | | | |
| 741-760 | PAPQAPYQGYQEPPAPQAPY | DR1/DR4 | 4th Q>P | 4th Q>P | 4th Q>P | 4th Q>P |
| | PAPQAPYQGYQEPPAPQAPY | DR1/DR4 | 8th Q>R | 8th Q>R | 8th Q>R | 8th Q>R |
| | PAPQAPYQGYQEPPAPQAPY | DR1/DR4 | 17th Q>P | | 17th Q>P | 17th Q>P |
| **LMP1 (386aa)** | | | | | | |
| 181-206 | LIWMYFHGPRHTDEHHHDDS | DR16 | 6th F>Y | 6th F>Y | 6th F>Y | 6th F>Y |

**Table S6. List of primers used for joining contigs**

| Primer sequence (5'-3') | Product size | Coordinates |
|---|---|---|
| TCATATGCTGACTGTATATGC | 1142 | 7426-8567 |
| GGGCAACACATAATCCTAGTG | | |
| TGTGGTTGGGCAGGTACATGC | 1192 | 36144-37335 |
| CTGGTCTCCATGGTCCACCGG | | |
| CAAGTTGCATTGGCTGCAAAG | 993 | 95891- 96883 |
| GGTGGAAAAATGGCCTTCTAC | | |
| CGATATACCTAGTGGGTGGAG | 712 | 40786-41497 |
| GCAACTCCGGGGCTGATCAG | | |
| TGGGTCAGACAGTTTGGTGCG | 768 | 147533-148300 |
| CCAATGACAAGCTTCCAGGCC | | |
| GACCTTGAGAGGGGCCCAC | 1300 | 167707-169007 |
| CATAGTAGCTTAGCTGAACTGG | | |
| TCATGTCTGACGAGGGGCCA | 1958 | 95660-97618 |
| TACGATTGAGGGCGTCTCCT | | |

*Coordinates of NC_007605
